# “Let's Talk About Your Note”: Using Open Notes as an Acceptance and Commitment Therapy Based Intervention in Mental Health Care

**DOI:** 10.3389/fpsyt.2021.704415

**Published:** 2021-07-19

**Authors:** Colin M. Smith, Alissa Stavig, Peter McCann, Ashley A. Moskovich, Rhonda M. Merwin

**Affiliations:** ^1^Department of Psychiatry and Behavioral Sciences, Duke University School of Medicine, Durham, NC, United States; ^2^Department of Medicine, Duke University School of Medicine, Durham, NC, United States

**Keywords:** acceptance and commitment therapy, open notes, mental health, experiential therapy, note writing

## Introduction

The United States (U.S.) 21st Century Cures Act “Open Notes Rule” took effect on April 5, 2021, requiring healthcare organizations to provide patients with immediate access to their clinical notes (1). The rule is the result of a 50-year movement to increase patient access to healthcare data (2, 3). Despite the fact that many healthcare organizations have been providing open notes to patients for over a decade, the release of mental health records has been less widespread (4). Mental health clinicians are now in a position to take stock of their note writing practices and consider implications for patient care. Here, we briefly review experiences with open notes to date, and, through the lens of Acceptance and Commitment Therapy (ACT), describe how open notes could serve as a therapeutic tool for mental health clinicians.

## A History of Open Notes and Implications for Mental Health Care

In 2010, a group of 100 primary care physicians were recruited as part of quasi-experimental study to evaluate open notes in three regions in the U.S. In this study, patients found open notes to be clinically relevant and 99% of patients wanted the practice to continue, while no physicians elected to stop using open notes (5). The U.S. The Department of Veterans Affairs was the first large U.S. health system to grant access to clinical notes for all patients, providing 1 million veterans access to their clinical notes in 2013 alone (6). With the help of open notes consortia (2), more than 50 million patients in the U.S. had access to their clinical records by 2020, but far fewer had access to mental health notes (7) despite calls for increasing this practice (8). At least 10 countries had provided patients at least some access to open notes as of 2018 (9).

Prior studies have reported clinician concerns about detrimental effects to the therapeutic relationship, patient well-being and workflow as a result of using open notes (6, 10–12). However, early data has found that patients who have access to their mental health notes requested information less often, attended more appointments and reported greater activation and recovery scores (13).

Advocates have framed open notes as a tool to increase patient autonomy, reduce stigma, empower patients to manage their mental health and strengthen the patient-clinician relationship (8, 12, 14). Use of open notes in practice has varied, with some providers using a passive approach (e.g., waiting for patients to ask about their notes), and others using a more active approach (e.g., encouraging feedback from patients) (12, 15). Patients have reported improved trust when providers use an active approach to discuss open notes, especially when notes were accurate, reflected details about the patient and highlighted strengths and progress (15).

There are broader complex implications of open notes on the therapeutic process. Open notes represent a significant shift from historical psychodynamic approaches to psychotherapy, where patients often did not see their practitioner, let alone their practitioner's description of their behaviors in concrete terms (16). While there has been movement away from this type of blinding, open notes move further down this continuum. This transition toward the patient “seeing the practitioner, seeing them” comes with the benefit of transparency and openness, but does have the added risk of patients feeling more vulnerable, exposed and medicalized. Even within cognitive-behavioral therapy (CBT), which has taken a straightforward, collaborative approach, sharing therapeutic process notes or documentation has not been typical. A related, but separate issue within behavior therapy is the awareness that the process of naming influences behavior. Open notes are likely to name patterns of behavior and contingencies influencing behavior, a core element of self-awareness and behavior change (17). Finally, patients knowing that information they relate in sessions will be conveyed in a note may also influence what they report or even their future behavior. These contingencies are always present in clinical interactions (18) but might be heightened in this new context. Below we describe how open notes may be used to augment therapeutic interventions, or explicitly leveraged as clinical intervention, using ACT as an example. Table 1 describes an example of an open note using ACT processes.

**Table 1:** Example of ACT processes in an open note.

**Brief case overview and clinical context**
Jane presents for the treatment of pregnancy anxiety in the context of previous miscarriage. Recent therapy sessions have focused on changing her relationship to difficult thoughts/feelings in the service of increasing meaningful life engagement (e.g., being fully present and engaged with her 4-year-old daughter during playtime even while feeling anxious).
**Documentation**
**Note Excerpt 1:** Today's session focused on helping Jane slow down, step back and notice her thoughts/feelings, rather than automatically reacting to them, by practicing the “I notice…” exercise (e.g., “I notice that I'm feeling anxious, I notice that my heart is racing, I notice I'm having the thought “something is wrong with my baby”). **ACT Process: function of documentation** •*Acceptance:* reading about the specific thoughts/feelings she described in session provides another opportunity for her to interact with these difficult experiences in new way. •*Cognitive defusion:* labeling the thought (i.e., I notice I'm having the thought that) and using quotation marks helps the client observe the thought as a product of the mind, decreasing its influence on behavior. •*Present moment awareness:* naming practice of moment-to-moment awareness supports skill acquisition and generalization. •*Self as context:* using observer language reinforces that the patient is separate and distinct from her experiences. **Sample experiential exercise utilizing open notes:** •Patient and clinician practice “I notice…” exercise while reviewing a note together. The clinician cues observing momentary experience with openness and curiosity (e.g., “What thoughts/feelings arise as you read this part of the note?”)
**Note Excerpt 2:** Discussed Jane's value of being a mother who “fully shows up” for her daughter and reviewed homework (committed action) to spend longer periods of time more fully engaged with her daughter during playtime, and less time in her head focused on her worries or unnecessarily checking fetal heart rate. Jane reports that while this was challenging, acknowledging and making space for anxiety (rather than fighting it) helped her spend more time with her daughter and check the monitor less frequently. **ACT Process: function of documentation** •*Acceptance:* describing positive consequences associated with allowing difficult thoughts/feelings reinforces acceptance. •*Values:* focusing on values helps the client orient toward valued-living rather than anxiety reduction and increases willingness to experience difficult thoughts/feelings by placing doing so in a frame of coordination with something that is important and meaningful. •*Committed Action:* stating the client's committed action reviewing progress reinforces behavior change and invites opportunity for reflection and growth. **Sample experiential exercise utilizing open notes:** •The clinician and patient practice noticing what it is like to notice herself behaving in a way that is consistent with her values (e.g., “What this like for you, to see yourself being the mom you want to be? Not perfectly, we are never perfect in our intentions, but moving with conviction in that direction?”).

## Open Notes as an Act Based Intervention in Mental Health Care

### Acceptance and Commitment Therapy

ACT, a third wave CBT developed in the 1980s by Hayes et al. (19), argues that the core of human suffering is psychological inflexibility—or the inability to be in the present moment with one's thoughts and feelings, and behave in a way that is consistent with personal values (19–22). This is exemplified in the rigid dominance of (1) experiential avoidance, (2) cognitive fusion, (3) conceptualized past and feared future, (4) attachment to the conceptualized self, (5) lack of values clarity, and (6) inaction. ACT increases psychological flexibility through six corresponding evidence-based processes of change: (1) acceptance, (2) cognitive defusion, (3) present moment awareness, (4) self as context, (5) values, and (6) committed action (20–22).

Open notes may be leveraged in several ways within an ACT intervention. Most globally, ACT is a densely experiential therapy, where processes, such as acceptance of unwanted feelings, are engaged in-the-moment in session (19, 22). Open notes can be used to provide a verbal description of these in-session experiences. When experiences are described verbally, they may be better retrieved from memory and repeated in daily life. Describing experiences that occurred in session may also provide an opportunity for the clinician to frame therapeutic experiences in terms of the individual's goals.

### Acceptance

Acceptance is the active process whereby individuals embrace difficult private experiences without attempts to rigidly avoid or control them (19, 22). Open notes may be used to reinforce acceptance, by documenting instances in which the patient engaged in acceptance behavior over the previous week, and specifically highlighting the positive consequences (i.e., how acceptance of unwanted feelings decreased the patient's suffering or increased their capacity to engage in their life in a meaningful way). This might be particularly useful for some patients who may miss verbal reinforcement in a face-to-face encounter or experience reinforcement as more salient in writing. A clinician might also use open notes to facilitate acceptance by encouraging a patient to review their session note, which contains difficult private content, and practice an open and willing stance. Patients may also be invited to practice openness to thoughts and feelings that arise as they review the note or notice if they have an urge to avoid this experience.

### Cognitive Defusion

Cognitive defusion is the process of changing the degree to which thoughts or other mental activity exert undue influence over behavior. Defusion promotes experiencing thoughts as products of the mind, rather than the literal events they represent (19, 22). Open notes can augment defusion by providing an opportunity for patients to see thoughts in writing, potentially creating distance between the patient and their internal experiences, so that they can observe them with less involvement. In order to encourage a defused stance and to decouple thoughts from actions, clinicians may put thoughts in quotes and identify a thought as being cued in a particular context (e.g., “Patient reports having the thought ‘I am lazy’ when allowing himself to take a break this past week”). Clinicians could also use notes to document specific defusion exercises completed in session (e.g., adding the clause “I notice I'm having the thought that…” to evocative thoughts) so that patients can continue to practice at home, or even ask patients to print notes to practice defusion (e.g., patients can put their note in their pocket to practice “carrying” difficult private events with them while they move in a valued direction) (23).

### Present Moment Awareness

Being present involves developing broad and flexible attentional processes. This includes taking in an array of information and attending to the here and now, rather than narrowly focusing toward or away from threat cues or on the conceptualized past or feared future (19, 22). Open notes could serve as an exercise in flexible attention, with the clinician encouraging the patient to observe the note in its entirety, rather than fixating on one aspect of the note to the neglect of others. Open notes might also provide an opportunity for patients to practice moment-to-moment awareness of the thoughts and feelings that arise while reading their note (or thinking about reading them) or notice if their mind travels to the past or future when interacting with the content.

### Self as Context

Self as context facilitates an observer stance (i.e., taking the perspective of the observer of one's experience), and is the antithesis of rigid or excessive attachment to the conceptualized self or “self-concept.” Self as context interventions encourage individuals to distinguish themselves from the content of their thoughts and feelings, and experience internal events as dynamic and changing elements of consciousness (19, 22). Reading open notes may allow the patient to take the perspective of the clinician or view themselves and their experiences from a different vantage point. Reading about one's thoughts and feelings may also help patients experience themselves as separate and distinct from experiences, which can be observed. Further, clinician notes may describe patients' behaviors in terms of the historical and situational context in which they emerge to enhance patient perspective on the factors that influence their behavior. Finally, open notes may also be used to decrease attachment to specific self-content, such as a diagnosis. Clinicians can use notes to guide patients into an awareness that they are separate or more than their diagnosis or history. This is accomplished by using more descriptive language of behavior and the use of person-centered language.

### Values

The goal of ACT is to help patients consciously connect with their values, which are chosen qualities of an action, and use these values to guide actions (19, 22). Open notes may be used to concretely document patients' stated values and facilitate reflection on whether they are living in accordance with those values. Clinicians might also choose to imbed a self-report scale, such as the valuing questionnaire (24), in their notes as a reference for patients. Notes can be used to reinforce working for values instead of reduction of momentary emotional pain.

### Committed Action

Committed action is about behaving in accordance with one's chosen values (19, 22). Concrete examples of committed actions aligned with values may be listed in the note to facilitate patients engaging in these actions or tracking their progress. Open notes may be used in order to document evidence-based behavioral interventions that support committed actions, such as goal setting, action planning, and problem-solving. Open notes may also be used to reinforce committed actions by documenting the positive steps that patients are taking, even if small. Finally, open notes may be used to document homework, or intentions to engage in committed actions that patients set for themselves. This may increase the likelihood that patients will follow through with assignments. Finally, open notes may be used to clarify the difference between patients' chosen values (life direction) and committed actions (goals that align with values).

## Limitations

In certain clinical contexts, such as individuals experiencing active psychosis or in cases of domestic abuse, open notes should not be used as an intervention. Indeed, the Cures Act supports withholding notes from patients when doing so would substantially reduce a risk of harm to a patient or others (1). There might also be instances where open notes could prove detrimental to patient experiential learning, such as in cases of where patients demonstrate increased pliance or when patient develop an overreliance on note content leading to reduced experiential learning. As in other therapeutic contexts, such clinically relevant behavior could be used as behavioral targets in therapy if identified and discussed openly. Since ACT is based in the pragmatic truth criterion, the workability of any particular behavior is paramount (19).

## Conclusions

Open notes are now the norm in mental health care in the U.S. Despite clinician concerns, early experiences suggest that patients and clinicians stand to benefit from embracing this change. Future studies should investigate using open notes as a clinical intervention while incorporating core processes of experiential therapies. ACT provides a framework for using open notes as a clinical tool in mental health care.

## Author Contributions

CS, AS, PM, AM, and RM conceptualized the manuscript. CS, AS, and PM reviewed the literature. CS wrote the initial outline of the manuscript. AS, PM, AM, and RM contributed to sections on the manuscript, reviewed and revised drafts of the manuscript, and provided conceptual guidance throughout. AM created Table 1 in the manuscript. All authors contributed to manuscript revision and reviewed and approved of the final version of the manuscript.

## Conflict of Interest

The authors declare that the research was conducted in the absence of any commercial or financial relationships that could be construed as a potential conflict of interest.
